# Clinical implication of cellular vaccine in glioma: current advances and future prospects

**DOI:** 10.1186/s13046-020-01778-6

**Published:** 2020-11-23

**Authors:** Yuanliang Yan, Shuangshuang Zeng, Zhicheng Gong, Zhijie Xu

**Affiliations:** 1 Department of Pharmacy, Xiangya Hospital, Central South University, 410008 Changsha, Hunan China; 2 National Clinical Research Center for Geriatric Disorders, Xiangya Hospital, Central South University, 410008 Changsha, Hunan China; 3 Department of Pathology, Xiangya Hospital, Central South University, 87 Xiangya Road, Hunan 410008 Changsha, China

**Keywords:** glioma, cellular vaccine, CART, DC vaccine, tumor lysate vaccine, immunotherapy

## Abstract

Gliomas, especially glioblastomas, represent one of the most aggressive and difficult-to-treat human brain tumors. In the last few decades, clinical immunotherapy has been developed and has provided exceptional achievements in checkpoint inhibitors and vaccines for cancer treatment. Immunization with cellular vaccines has the advantage of containing specific antigens and acceptable safety to potentially improve cancer therapy. Based on T cells, dendritic cells (DC), tumor cells and natural killer cells, the safety and feasibility of cellular vaccines have been validated in clinical trials for glioma treatment. For TAA engineered T cells, therapy mainly uses chimeric antigen receptors (IL13Rα2, EGFRvIII and HER2) and DNA methylation-induced technology (CT antigen) to activate the immune response. Autologous dendritic cells/tumor antigen vaccine (ADCTA) pulsed with tumor lysate and peptides elicit antigen-specific and cytotoxic T cell responses in patients with malignant gliomas, while its pro-survival effect is biased. Vaccinations using autologous tumor cells modified with TAAs or fusion with fibroblast cells are characterized by both effective humoral and cell-mediated immunity. Even though few therapeutic effects have been observed, most of this therapy showed safety and feasibility, asking for larger cohort studies and better guidelines to optimize cellular vaccine efficiency in anti-glioma therapy.

## Background

Arising from supporting glial cells, gliomas represent over 36% of malignant primary central nervous system (CNS) tumors [1]. Gliomas vary in aggressiveness from low-grade to highly malignant, with 5 year overall survival no greater than 35% [2]. According to the pathological features and clinical outcomes, the World Health Organization (WHO) grades gliomas on a scale of I to IV. The most benign brain tumor is designated grade I, has distinct boundaries, grows slowly, rarely spreads and typically arises in childhood. The most common glioma in adults is glioblastoma (GBM), an astrocytoma designated by the WHO as grade IV. GBMs remain among the most difficult brain tumors to treat, with a median survival of less than 2 years, despite surgical resection, radiation, and chemotherapy [3]. Over the past decades, an explosion in the understanding of treatment strategies of gliomas has progressed beyond the standard of care and consists of complete resection followed by radiotherapy and pharmacological treatment with chemotherapeutic agents, such as temozolomide. For example, on the basis of mutations in the genes encoding the isocitrate dehydrogenases IDH1 and IDH2, co-deletion of 1p19q (oligodendroglioma) and methylation of O^6^-Methylguanine-DNA methyltransferase (MGMT) have been subclassified as molecular diagnostics and prognostic markers for gliomas [4]. Unfortunately, limited therapeutic access to brain tumor and peritumoral tissue caused by the blood-brain barrier (BBB) still offers a new challenge to optimize glioma treatment, despite such efforts [5]. Considering the special anatomical position of gliomas, new cancer therapies need to be discovered to achieve optimally safer, less invasive and more effective treatment. On the basis of these observations, the clinical success of immunotherapy seems a predictable option in basic biological principles of glioma diagnosis and prognosis.

Cancer immunotherapy, also known as immuno-oncology, is a type of cancer treatment that uses the power of the body’s own immune system to prevent, control, and eliminate cancer [6, 7]. Recently, immunotherapy has been proposed against existing neoplasms, including breast cancer, lung cancer and even glioma, and may show significant promise where other approaches have failed [8]. Based on immune checkpoint inhibitors and vaccine-mediated immunization, previous studies have reported immunotherapeutic strategies that reverse immunosuppressive tumor environment, stimulate antigen presentation, and induce anti-tumor T cell responses [9]. For example, the dendritic cell (DC)-based vaccinations has been established as a promising approach for the immunotherapy of cancer [10–12]. In tumor cells, the physical modalities inducing immunogenic cell death (ICD), such as radiotherapy, UV light C, high hydrostatic pressure (HHP), hypericin-based photodynamic therapy (Hyp-PDT) or hyperthermia, are of particular interest in the development of DC-based vaccines for cancer immunotherapy [13]. Using an orthotopic HGG murine model, Garg et al.. observed that DC-based vaccines pulsed with Hyp-PDT-induced ICD provided strong anti-glioma survival benefit by inducing an immunostimulatory shift in the brain immune contexture from T regulatory cells (Tregs) to T helper 1 (Th1)/cytotoxic T lymphocyte (CTL)/Th17 cells. Meanwhile, a combination of ICD-based DC vaccines and temozolomide synergistically prolonged overall survival in malignant glioma-bearing mice, leading to ~ 50% long-term survivors [14]. Thus, therapeutic vaccines represent another valuable option for the management of glioma immunotherapy. Moreover, the demonstration of the efficacy of a vaccine for gliomas seems relatively straightforward to improve both anti-tumor innate and adaptive immune responses.

**Fig. 1:**
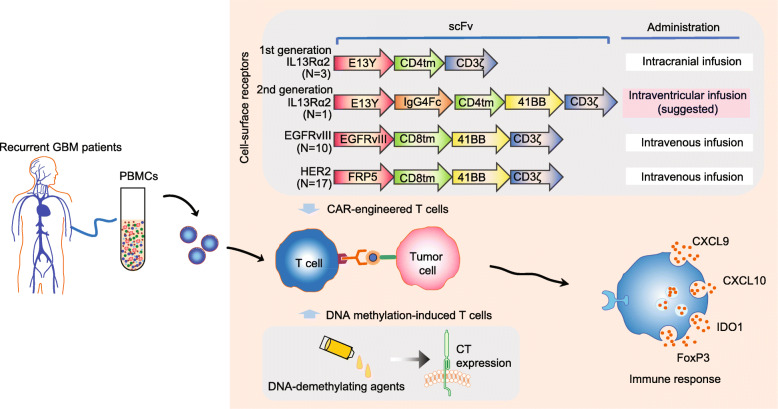
Cellular vaccine with TAA-engineered T cells in anti-glioma clinical trial. In glioma treatment, particularly, recurrent GBM, therapy with TAA-engineered T cells mainly uses chimeric antigen receptors (CARs) and DNA methylation-induced technology. For CAR-engineered T cells, single-chain variable fragments (scFv) of cell-surface receptor-engineered T cells mainly include IL13Rα2, EGFRvIII and HER2 selective ligands. In addition, treatment with DNA-demethylating agents, such as 5-aza-2’-deoxycytidine, facilitates CT antigen expression and constitutes an attractive immunological target for cancer immunotherapy. E13Y, IL13Ra2-selective ligand IL13; FRP5, HER2-specific MAb; CD4tm, CD4 transmembrane domain; CD8tm, CD8 transmembrane domain; 4-1BB, CD137 cytoplasmic signaling domain; CD3ζ, cytoplasmic signaling domain sequences

Surprisingly, cellular vaccines, which can be divided into autologous and allogeneic vaccines, have achieved significant levels of objective response in glioma treatment [15]. Theoretically derived from the patient’s own tumor, autologous vaccines have numerous potential advantages. The most important benefit is that autologous vaccines are likely to carry the unique tumor-associated antigens (TAA), which have been investigated as the specific immunological targets for immunotherapy in particular patients [16]. In a phase I/II trial in patients with recurrent malignant glioma, a DC-based multi-peptide vaccine derived from glioma-associated antigens (GAA) showed expected clinical efficacy in 22 patients, about 41% of vaccinated patients remained progression-free for ≥ 12 months [17]. More importantly, the safety and measurable immune response of the autologous ICT-107 vaccines produced from TAA-pulsed DC were confirmed for patients with newly diagnosed GBM in a phase I clinical trial [18]. However, even though vaccine efficacy has been demonstrated, the difficulty to produce and harvest enough vaccine doses still exists and diminishes its ultimate availability for clinical usage as an autologous vaccine. Unlike autologous cell therapy, allogeneic vaccines are composed of intact or modified cells harboring shared antigens found on a large percentage of gliomas from other patients or healthy donors. Chimeric antigen receptors (CARs) are artificial fusion proteins that incorporate an extracellular ligand recognition domain, a transmembrane domain, and an intracellular signaling domain to induce T cell activation upon antigen binding [19, 20]. Genetic engineering of allogeneic T cells to express CARs directed against specific antigens has explored a new way to optimize personalized glioma therapy [21]. In this review, we summarize the safety, feasibility, and immune/tumor response of cellular vaccines based on T cells, DC cells, tumor cells and natural killer (NK) cells in preclinical and clinical trials for glioma immunotherapy, and discuss the future prospects to optimize vaccine-mediated immunity according to current findings (Fig. 1).

### Current strategy of cellular vaccine in glioma immunotherapy

High-grade glioma, especially recurrent GBM, is almost inevitable, and the prognosis remains poor, with a median survival of 12–15 months [22]. Immunotherapies using autologous vaccines are now considered promising approaches for treating patients with malignant gliomas. The microenvironment of brain tumors contains unique tissue-resident cell types, in addition to cancer cells, including microglia, astrocytes, neurons and immune cells. The normal brain is considered an “immune privileged” organ that is sheltered from immune cell entry through the integrity of the BBB, which physically protects the brain from inflammatory factors that can be cytotoxic and cause neurodegeneration [23, 24]. On the other hand, peripheral immune cells infiltrating from the circulation can cross the BBB and enter the CNS after injury and, in disease, then join resident immune cells to modulate neuroprotective lymphocyte responses and brain function via their interactions with glia [25]. Indeed, current data indicate that therapeutic vaccines based on T cells, DC cells, tumor cells and NK cells are feasible and generally well tolerated. Furthermore, their clinical efficacy has been demonstrated in several randomized clinical trials.

### TAA engineered T cells

Adoptive T cell transfer can overcome the in vivo progression of gliomas by using autologous cells with engineered receptor specificities for TAAs [26]. Antigen-specific T cells can be expanded *ex vivo* for subsequent administration, which produces the cytokines that are essential for T cell expansion and sustained anti-tumor activity [27]. CAR-engineered T cell (CAR T cell) therapy is a promising therapeutic approach genetically generated with modified T cells to express recombinant protein CARs that may be effectively and safely applied to GBMs to reduce recurrence rates [28, 29]. Several cell surface proteins, such as interleukin 13 receptor α2 (IL13Rα2), epidermal growth factor receptor variant III (EGFRvIII), ephrin type-A receptor 2 (EphA2), and human epidermal growth factor receptor 2 (HER2), have been found to actively target CAR T cell therapy in preclinical models [30–33], but only a few of these cell-surface receptors have been validated in clinical trials. Accordingly, a phase I/II clinical study of adoptive immunotherapy suggests that anti-EGFRvIII CAR-engineered T cells effectively produced the effector cytokines and interferon-γ, contributing to lyse the antigen-expressing glioma cells [34]. Meanwhile, another completed phase I clinical trial program (NCT01109095) reveals that anti-HER2 CAR CMV-specifc T cells seem to be able to inhibit HER2 + glioma growth [35]. Here, to improve anti-glioma responses, we discuss the use of TAA-engineered T cells through their clinical strategies and outcomes under investigation.

### IL13Rα2-engineered T cells

IL13Rα2, a cell-surface receptor positively expressed in 82% of GBM samples and > 70% of glioma stem-like cancer initiating cells [36, 37], was previously thought to be directly associated with increased mesenchymal signature gene expression and poor patient survival [38]. For the treatment of recurrent GBM, Christine et al. showed the first-in-human clinical experience for CAR-engineered IL13Rα2-specific CD8^+^ CTL and observed significant tumor regression. Briefly, for autologous IL13-zetakine^+^ CD8^+^ CTL manufacturing, the peripheral blood mononuclear cells (PBMCs) were stimulated with anti-CD3 antibody, followed by DNA electroporation, drug selection and ex vivo expansion using OKT3 and irradiated feeders. In three patients with recurrent GBM, the feasibility of repetitive intracranial administration of first-generation IL13Rα2-specific CD8^+^ CAR T cells was demonstrated and transient anti-tumor activity for some patients was reported in the absence of serious adverse events, such as occlusion, malfunction, or infection [30]. Building on these results, the modified IL13Rα2-targeted CAR T cells were further reported to improve anti-tumor potency and T cell persistence by 4-1BB co-stimulation and IgG4-Fc linker mutation [39]. A patient with recurrent multifocal GBM who received treatment with modified IL13Rα2-targeted CAR T cells had regression of all intracranial and spinal tumors, along with significant increases in the levels of cytokines C-X-C motif chemokine ligand 9 (CXCL9) and CXCL10, as well as immune cells in the cerebrospinal fluid [28]. Comparing the ability to abrogate tumor growth at local and distant sites, Christine et al. suggested intraventricular administration of CAR T cells is better than intracavitary therapy for the treatment of malignant brain tumors. Nevertheless, the above evidence of the safety and anti-tumor activity of IL13Rα2-targeted CAR T cell immunotherapy still needs to be evaluated in a larger cohort of patients.

### EGFRvIII-engineered T cells

Negative prognostic indicator EGFRvIII is expressed in about 25–33% of all patients with GBMs [40] and is the most commonly mutated gene among the EGFR family in glioma [41]. In EGFRvIII-expressing newly diagnosed GBM, a peptide vaccine targeting EGFRvIII (rindopepimut) was previously evaluated and found to be well tolerated, providing immune responses with prolonged progression-free survival [42, 43]. More recently, O’Rourke et al. conducted a phase I safety study of autologous CAR T cells targeted to EGFRvIII (CART-EGFRvIII) in 10 patients with recurrent GBMs. Intravenous infusion of a single dose of CART-EGFRvIII cells was found to be feasible and safe, without off-tumor toxicity or cytokine release syndrome [44]. For vaccine delivery, CART-EGFRvIII cells were detected transient expansion in peripheral blood. Trafficking of CART-EGFRvIII cells were also found in regions of active GBM in 7 patients with surgical intervention. Compared to pre-CART-infusion, tumors had markedly induced expression of immunosuppressive molecules (IDO1 and FoxP3) post-infusion. However, marked tumor regression was not observed by MRI over 18 months of follow-up after CART infusion. It is possible that this invalid clinical benefit of CART-EGFRvIII, which can be definitively determined from a large sample size, may improve the responsiveness of tumors through intraventricular therapy.

### HER2-engineered T cells

Elevated HER2 expression has been reported in 41% of primary GBM samples and in 81.4% of GBM primary cell lines and were correlated with impaired survival [45, 46]. In preclinical models of GBM, bispecific CAR molecules that incorporated 2 antigen recognition domains for HER2 and IL13Rα2 showed the functional superiority of T cell expressing antigens *ex vivo* and in an orthotopic GBM xenograft model [47]. However, the safety concerns of autologous CART-HER2 cells were raised by the report of a serious adverse event following the administration 1 × 10^10^ T cells of vaccine based on trastuzumab [48]. While administration of up to 1 × 10^6^/m^2^ CART-HER2 cells showed no evident toxicities, unfortunately, the expansion and persistence of CART-HER2 cells was limited [49]. To treat HER2-positive GBM, Nabil Ahmed et al. developed HER2-specific CAR-modified virus-specific T cells (CAR VS Ts-HER2) with an FRP5-based exodomain and a CD28.ζ endodomain [50]. Up to 1 × 10^8^/m^2^ CAR VS Ts-HER2 were infused intravenously without dose-limiting toxic effects in 17 patients with progressive GBM. After the infusion, CAR VS Ts-HER2 was detectable in the peripheral blood for up to 12 months, with no observed expansion in peripheral blood (but expansion at GBM sites). Of 16 evaluable patients, 50% of patients had clinical benefit, as defined by a partial response (N = 1, over 9 months) and stable disease (*N* = 7, 8 weeks–29 months). Despite the feasibility and safety of CAR VS Ts-HER2, a clinical strategy using it alone or in combination with other immunomodulatory approaches is warranted to improve the anti-GBM activity of CAR VSTs-HER2 by augmenting their expansion and persistence.

### DNA methylation-induced T cells

In cancer cells, epigenetic regulation by DNA methylation facilitates cancer/testis (CT) antigen expression and constitutes an attractive immunological target for cancer immunotherapy [51–53]. Recently, a novel approach was reported based on the adoptive transfer of cytotoxic lymphocytes with specificity for antigens induced by a DNA demethylating agent [54]. In this report, Kirkin et al.. found that after treatment with 5-aza-2’-deoxycytidine, a DNA-demethylating agent, activated CD4^+^ T helper cells from the patients with recurrent GBM could express a broad repertoire of endogenous CT antigens and serve as antigen-presenting cells to generate autologous CTL and NK cell responses. This group also revealed that injections of tumor-reactive lymphocytes generated by this DNA demethylation-mediated procedure into peripheral vein of GBM patients generated persistent anti-tumor immune response without treatment-related adverse effects. Treatment of cancer cells with 5-aza-2’-deoxycytidine also leads to the increased levels of MHC and costimulatory molecules (CD80, CD86, and CD40) necessary for the antigen-presenting function of DC cells [55, 56]. In addition, as an important determinant of anti-tumor immune response, DNA methylation aberration implicates epigenetic modulation as a combination regimen for potential precision immunotherapy with checkpoint blockade [57].

Overall, TAA-engineered T cells, along with DNA-demethylated T helper cells against recurrent GBMs, initially confirm the safety and anti-tumor activity of autologous T cell immunotherapy, providing a minimally invasive strategy for glioma treatment and prevention.

### Autologous dendritic cell/tumor antigen vaccine (ADCTA)

**Fig. 2:**
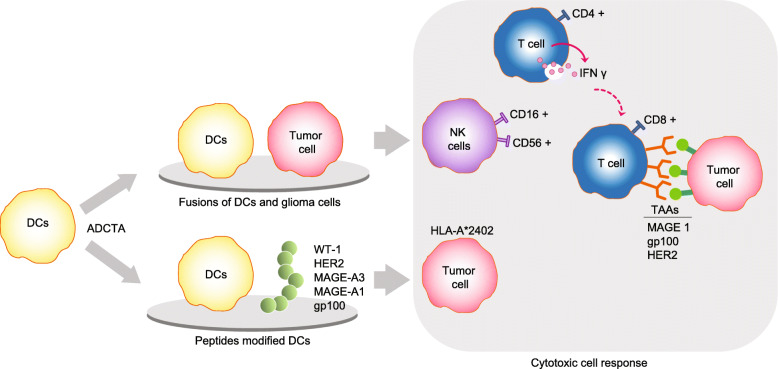
Autologous dendritic cell/tumor antigen vaccine (ADCTA) in anti-glioma clinical trial. Vaccination with tumor lysate-pulsed dendritic cells (DC) elicits antigen-specific, CD4^+^/CD8^+^ cytotoxic T cell responses and induces IFN γ secretion in patients with malignant glioma. The peptide modified DC with cocktail (WT-1, HER2, MAGE-A3, and MAGE-A1 or gp100) had a positively response in HLA-A24^+^ glioma patients

DC therapy is a safe and well tolerated immunotherapeutic method, and its clinical effectiveness has been validated in melanoma, prostate cancer, malignant glioma, and renal cell carcinoma [58]. Previous studies have demonstrated that microglia are the predominant antigen-presenting cells (APCs) in the brain [59], while DC vaccines are likewise gaining significant clinical attention as a complementary strategy to stimulate T cell responses [60]. For instance, DC derived from blood monocytes could further enhance tumor-specific CD8^+^ T cell polyfunctionality *in vivo* when administered as a vaccine [61]. Recently, the presence of human cytomegalovirus (HCMV) antigens has been specifically found in gliomas, but not surrounding normal brain tissue. These unique and immunogenic HCMV antigens provide an attractive opportunity to leverage HCMV antigens as tumor-specific immunotherapy targets whilst minimizing toxicity [62]. To prove this conjecture, a randomized pilot trial in patients with newly diagnosed GBM was conducted to demonstrate that HCMV pp65 RNA-loaded DC vaccination experienced enhanced tumor-specific CD8^+^ T cell polyfunctionality and significantly increased overall survival [63]. The initial results obtained from clinical trials of autologous antigen or peptide-pulsed DC appear to be encouraging for a variety of tumors [64, 65]. The clinical trials testing DC vaccines after modified or adjuvant treatment with novel chemotherapy for gliomas defined as malignant or special subtype are underway at the NIH Clinical Center (Table 1). Here, we mainly review the feasibility and safety of autologous DC vaccines in phase I-III clinical trials and discuss recent progress in immunotherapy using autologous DC-based vaccination (Fig. 2).

**Table 1:** Clinical trial of DC vaccine in glioma

Malignancy	Phase	Number	Study title	Identifier	Status	Interventions	Observation
**DC vaccine**							
Malignant glioma	I	35	Surgical resection with gliadel wafer followed by dendritic cells vaccination for malignant glioma patients (Gliadel Wafer)	NCT00576446	Completed	NA	PFS
Malignant glioma	I	8	Vaccine therapy in treating patients with malignant glioma	NCT00612001	Completed	GAA pulsed DC	Toxicity; PFS; OS
Recurrent malignant glioma	I/II	22	Vaccination-dendritic cells with peptides for recurrent malignant gliomas	NCT00766753	Completed	GAA pulsed DC	Toxicity; PFS; OS
Recurrent GBM	I	20	A Study of ICT-121 dendritic cell vaccine in recurrent glioblastoma	NCT02049489	Completed	CD133 pulsed DC	Toxicity; feasibility; PFS; OS
Recurrent GBM	I	50	Vaccine therapy in treating patients undergoing surgery for recurrent glioblastoma multiforme	NCT00890032	Completed	BTSC mRNA-loaded DC	Toxicity; feasibility; humoral and cellular immune responses
Malignant glioma	I	71	Dendritic cell vaccine with imiquimod for patients with malignant glioma	NCT01792505	Completed	Imiquimod adjuvant	Toxicity; PFS
Malignant glioma	I	6	Nivolumab with DC vaccines for recurrent brain tumors (AVERT)	NCT02529072	Completed	Nivolumab adjuvant	Toxicity; PFS; OS
IDH1(R132H) mutant glioma	I	30	Safety and efficacy of IDH1 R132H-DC vaccine in gliomas	NCT02771301	Recruiting	IDH1(R132H) DC	IDH1(R132H) specific T cells; anti-IDH1(R132H) antibody level
Newly diagnosed GBM	I	10	Personalized cellular vaccine for glioblastoma (PERCELLVAC)	NCT02709616	Active, not recruiting	TAA pulsed DC	Toxicity; PFS; OS; CD8 + and CD4 + T cell response
Recurrent GBM	I	10	Personalized cellular vaccine for recurrent glioblastoma (PERCELLVAC2)	NCT02808364	Active, not recruiting	TAA pulsed DC	Toxicity; PFS; OS; CD8 + and CD4 + T cell response
HGG	I	11	Cytomegalovirus (CMV) RNA-pulsed dendritic cells for pediatric patients and young adults with WHO grade IV glioma, recurrent malignant glioma, or recurrent medulloblastoma (ATTAC-P)	NCT03615404	Active, not recruiting	CMV RNA-pulsed DC	Toxicity
Pediatric HGG	I	8	Adoptive cellular therapy in pediatric patients with high-rade gliomas (ACTION)	NCT03334305	Recruiting	GM-CSF and TTRNA-xALT plus Td vaccine without autologous HSCs	Toxicity; PFS; OS; INF γ secretion
DIPG	I	21	Brain stem gliomas treated with adoptive cellular therapy during focal radiotherapy recovery alone or with dose-intensified temozolomide (Phase I) (BRAVO)	NCT03396575	Recruiting	GM-CSF and TTRNA-xALT plus Td vaccine without autologous HSCs	Toxicity; feasibility; PFS; OS; immune responses
GBM; anaplastic astrocytoma	I	20	Dendritic cell (DC) vaccine for malignant glioma and glioblastoma	NCT01808820	Active, not recruiting	Post-DC Vaccine therapy, administered lysate of tumor	Toxicity; PFS; OS; MDSC levels and blood counts
Newly diagnosed GBM	I	42	Vaccine therapy in treating patients with newly diagnosed glioblastoma multiforme (ATTAC)	NCT00639639	Active, not recruiting	pp65-shLAMP DC and Td	Toxicity; feasibility; PFS; OS; humoral and cellular immune responses
GBM	I	39	Phase I study of a dendritic cell vaccine for patients with either newly diagnosed or recurrent glioblastoma	NCT02010606	Active, not recruiting	Bevacizumab adjuvant	Toxicity; PFS; OS; cytotoxic T cell activity; tumor stem cell antigen expression
GBM; DIPG	I	10	Immune modulatory DC vaccine against brain tumor	NCT03914768	Enrolling by invitation	Cyclophosphamide and bevacizumab adjuvant	Toxicity; tumor response; PFS; OS
GBM	I/II	10	Dendritic cell vaccination for patients with solid tumors	NCT01291420	Enrolling by invitation	WT1 mRNA-electroporated DC	Immunogenicity
Pediatric glioma	I	1	Dendritic cell vaccine therapy with in situ maturation in pediatric brain tumors	NCT01902771	Terminated	Post-Vaccine, lysate of tumor and imiquimod adjuvant	Toxicity; feasibility; PFS; OS; immune response
Medulloblastoma; PNET	I/II	1	Phase I/II: decitabine/vaccine therapy in relapsed/refractory pediatric high grade gliomas/medulloblastomas/CNS PNETs	NCT02332889	Terminated	GM-CSF and Poly-ICLC adjuvant	Tolerability
Newly diagnosed GBM	II	11	Phase II Feasibility Study of Dendritic Cell Vaccination for Newly Diagnosed Glioblastoma Multiforme	NCT00323115	Completed	NA	Toxicity; tumor response; PFS; OS; T Cell immune responses
Newly diagnosed GBM	II	120	Vaccine therapy for the treatment of newly diagnosed glioblastoma multiforme (ATTAC-II)	NCT02465268	Recruiting	pp65-shLAMP DC with GM-CSF and Td	PFS; OS; cytokine
Newly diagnosed MGMT unmethylated HGG	II	48	Immunotherapy targeted against cytomegalovirus in patients with newly-diagnosed WHO grade IV unmethylated glioma (I-ATTAC)	NCT03927222	Recruiting	pp65-shLAMP DC with GM-CSF and Td	Toxicity; migration; OS
GBM	II	100	Study of DC vaccination against glioblastoma	NCT01567202	Recruiting	Glioma stem-like cells (A2B5+) loaded DC	Tumor response; PFS; OS
Primary GBM	II	24	A Phase II, randomized, Open-Label, parallel-group study to evaluate the efficacy and safety of autologous dendritic cell vaccination (ADCV01) as an Add-On treatment for primary glioblastoma multiforme (GBM) patients	NCT04115761	Recruiting	NA	PFS
GBM	II/III	60	Dendritic cell immunotherapy against cancer stem cells in glioblastoma patients receiving standard therapy (DEN-STEM)	NCT03548571	Recruiting	NA	PFS; OS; immune responses
**ADCTA**							
Malignant glioma	I	28	Vaccine therapy in treating patients with malignant glioma	NCT00068510	Completed	NA	Toxicity; tumor response; OS; immune responses
Pediatric malignant glioma	I	7	Vaccine therapy in treating young patients who are undergoing surgery for malignant glioma	NCT00107185	Completed	NA	Toxicity; PFS; OS
Malignant glioma	I	8	Vaccination with dendritic cells loaded with brain tumor stem cells for progressive malignant brain tumor	NCT01171469	Completed	Imiquimod adjuvant	Toxicity; tumor response
Newly diagnosed GBM	I	21	Vaccine therapy and temozolomide in treating patients with newly diagnosed glioblastoma	NCT01957956	Active, not recruiting	NA	Toxicity; tumor response; PFS; OS
Recurrent GBM	I	20	Vaccine therapy in treating patients with recurrent glioblastoma	NCT03360708	Recruiting	NA	Toxicity; tumor response; PFS; OS
Recurrent GBM	I	40	Pembrolizumab and a vaccine (ATL-DC) for the treatment of surgically accessible recurrent glioblastoma	NCT04201873	Recruiting	Pembrolizumab and poly ICLC adjuvant	Toxicity; PFS; OS; Cell cycle-related signature; TIL density; TCR Clonality
GBM	I/II	10	Clinical study of a dendritic and glioma cells fusion vaccine with IL-12 for treatment-naïve GBM patients	NCT04388033	Recruiting	IL-12 adjuvant	Toxicity; PFS; OS
HGG	I/II	25	Autologous dendritic cells and metronomic cyclophosphamide for relapsed high-grade gliomas in children and adolescents	NCT03879512	Recruiting	Cyclophosphamide adjuvant	Toxicity; PFS; OS; Treg; CTL; cytokine
GBM	II	87	Dendritic cell cancer vaccine for high-grade glioma (GBM-Vax)	NCT01213407	Completed	Trivax adjuvant	PFS; OS
GBM	II	26	Efficacy & safety of autologous dendritic cell vaccination in glioblastoma multiforme after complete surgical resection	NCT01006044	Completed	NA	Toxicity; PFS; OS; humoral/cellular response
LGG	II	18	Vaccine for patients with newly diagnosed or recurrent low-grade glioma	NCT01635283	Active, not recruiting	NA	PFS; OS
GBM	II	50	Dendritic cell-based tumor vaccine adjuvant immunotherapy of human glioblastoma multiforme	NCT02772094	Active, not recruiting	NA	Toxicity; PFS; OS
Newly diagnosed GBM	II	136	Efficiency of vaccination with lysate-loaded dendritic cells in patients with newly diagnosed glioblastoma (GlioVax)	NCT03395587	Recruiting	NA	PFS; OS
Glioma	II	60	Dendritic cell vaccine for patients with brain tumors	NCT01204684	Active, not recruiting	Poly-ICLC adjuvant	PFS; OS

*DC* dendritic cell; *ADCTA* autologous DC/tumor antigen vaccine; *GAA* glioma-associated antigen; *GM-CSF* granulocyte macrophage-colony stimulating factor; *MAGE-A1* melanoma antigen gene-A1; *MAGE-A3* melanoma antigen gene-A3; *CMV* cytomegalovirus; *SAA* stem-like cells associated antigens; *TAA* tumor-associated antigens; *DIPG* diffuse intrinsic pontine glioma; *MDSC* myeloid derived suppressor cell; *Poly-ICLC* polyinosinic-polycytidilic acid; *GAAs* glioma-associated antigens; *PNET* primitive neuroectodermal tumor; *GBM* glioblastoma; *HGG* high-grade glioma; *LGG* low-grade glioma; *NA* not applicable; *Td* tetanus-diphtheria; *CTL* cytotoxic T cell; *HSCs* hematopoietic stem cells; *TCR T* cell receptor; *TIL* tumor infiltrating lymphocyte; *WT1* Wilms’ Tumor Gene

### Fusions of autologous DC and glioma cells

DC can sample tumor antigens through capturing the ‘eat me’ or ‘do not eat me’ signal of tumor cells [66]. Molecules such as milk fat globule-EGF factor 8 (MFG-E8) bridge the phosphatidylserine of dying cells with integrin αvβ3 of DC, then link to downstream signals through integrin receptors on the phagocyte [67]. Tumor lysates include poorly identified high-grade glioma-specific tumor antigens, which have practicality in terms of personalized medicine [68]. Previous studies have indicated that vaccination with glioma cell lysate-pulsed DC elicited a stronger specific CTL response, thereby preventing glioma formation in C57BL/6 mice model [69]. More importantly, combinatorial treatment of tumor lysate-pulsed DC vaccines and other therapeutic strategies, such as checkpoint inhibitors [70], angiogenesis inhibitors [71] and cytokine gene therapy [72], conferred a greater survival advantage and significantly increased the therapeutic anti-glioma efficacy. In addition, counteracting the immunosuppressive environment before vaccination is requisite to facilitate the long-term anti-glioma immune responses [9]. Nowadays, using DC from the peripheral blood of patients pulsed with an autologous tumor lysate is currently being evaluated in phase I/II clinical trials for gliomas [73], but the objective response rate still has been relatively low in some research.

Vaccination with tumor lysate-pulsed DC elicits antigen-specific and CTL responses in patients with malignant glioma. The percentage of NK cells, such as CD16^+^ and CD56^+^ cells, in peripheral blood lymphocytes were safely increased after immunization [73, 74]. Accordingly, a significant positive correlation was observed between activated NK cell populations and overall survival in patients administrated with autologous tumor lysate-pulsed DC vaccination [75]. For newly diagnosed GBM, autologous vaccination with tumor lysate-pulsed DC enhanced the precursor frequency of CD4^+^ T and CD4^+^ interferon (IFN) γ-producing cells, suggesting an induced tumor-specific CTL response [76]. A recent phase I study was conducted to evaluate the safety and bioactivity of vaccination with tumor lysate-pulsed DCs in patients with GBM and anaplastic astrocytoma. In response to tumor lysate after vaccination, most of patients displayed robust systemic cytotoxicity as indicated by peripheral IFN γ accumulation and intratumoral CD8^+^ T cell infiltrate. Furthermore, no clinical evidence of autoimmune diseases was detected, suggesting that tumor lysate-pulsed DC vaccination was safe [77, 78]. By comparing specific immune responses before or after vaccination, up-regulation and/or cytoplasmic accumulation of chemoresistance-associated peptides, including Wilms tumor protein (WT1), glycoprotein 100 (gp100), and MAGE family member A3 (MAGEA3), were noted in tumors being treated with immunotherapy [79]. Yu et al. noted significant expansion in CD8^+^ antigen-specific T cell clones against TAAs, including melanoma-associated antigen (MAGE) 1, gp100, and HER2, and the CD8^+^ T cell intratumoral infiltrate was increased in 50% of patients [77]. Moreover, tumor lysate and IL-18 loaded DC vaccines can elicit a specific CD8^+^ cytotoxic T lymphocyte response in GBM patients. The cytotoxic responses were augmented by transfecting DC with the gene for IL-18, but significantly inhibited by anti-human leukocyte antigen (HLA) class I antibody [80].

In addition, several small size studies showed biased results with or without a pro-survival effect from tumor lysate-pulsed DC vaccination in glioma patients. Ryuya et al. showed the maturation of DC with OK-432, granulocyte-macrophage colony-stimulating factor (GM-CSF) and IL-4 that were pulsed with autologous tumor lysate showed better survival in 24 patients with recurrent GBMs. Also, patients injected with both intratumoral and intradermal administrations had longer survival times as compared with intradermal administration only [81]. A phase II trial demonstrated 53% of GBM patients (*N* = 32) exhibited over a 1.5-fold increase in vaccine-enhanced cytokine (IFN γ) and had significantly longer times to tumor progression and survival [82]. Furthermore, intradermal administration of fusion cells and subcutaneous injection of recombinant human interleukin 12 (rhIL-12) at the same site showed a greater than 50% reduction in tumor size in some patients without adverse effects and safely induced clinical anti-tumor effects with malignant gliomas [83]. Accounting for the narrow therapeutic index of rhIL-12 [84], the alteration of chemoresistance-associated peptides may achieve susceptibility of TAA-expressing glioma cells to the specific immune response in DC-based immunotherapy. However, vaccination with Audencel, a tumor lysate-charged autologous DC vaccine, failed to improve progression-free survival and median overall survival, although no severe toxicity was observed in those newly diagnosed with GBM (*N* = 76) [85]. The factors dictating the efficacy of DC vaccines may represent a viable strategy to improve anti-tumor immunotherapy. The enzyme-linked immune absorbent spot (ELISPOT) is one of the most commonly used to understand the frequency of cytokine-secreting cells at the single-cell level [86]. Increased ELISPOT and delayed-type hypersensitivity responses after vaccination could provide good laboratory markers to predict the clinical outcome of patients receiving DC vaccination [73, 81]. The content of tumor-infiltrating lymphocytes (TILs) and T cell receptor (TCR) repertoire in brain tumors and peripheral blood have been proved to be correlated with improved clinical outcome in GBM patients [87]. TCR repertoires are widely shared sequences that have been suggested to be over-represented due to their potential immune functionality or their ease of generation by V(D)J recombination [88]. After treatment, tracking TCR repertoire shifts in tumors and peripheral blood can be used to monitor the treatment-associated immune response without the need to know the specificity of receptors [89]. Hsu and colleague revealed a statistically significant correlation between higher degrees of TCR repertoires and prolonged overall survival in autologous tumor lysate-pulsed DC vaccines-treated GBM patients [87]. In a prospective case control study that enrolled 47 GBM patients with DC vaccine adjuvant therapy, better outcomes were predicted with younger age and a lower programmed cell death protein 1 (PD-1)^+^/CD8^+^ ratio in TILs and PBMCs [90]. Further analysis of the immune system factors demonstrated that the patients with an immune system equipped with favorable pre-existing or post-vaccination anti-tumor capabilities, such as IFN γ secretion and CD8^+^ cells, are more likely to live longer [91]. Also, another clinical trial protocol of DC vaccination, including inclusion/exclusion criteria, was proposed and may increase the immune response and safety in pediatric and adult subjects [92, 93]. Thus, even DC immunotherapy against GBM has some exciting outcomes, and the bias caused by sample size, mode of administration and non-specificity of vaccine-target interactions indicate that investigating combination therapies or developing meaningful biomarkers should be studied in further phase II/III clinical trials.

Standard therapy for GBMs post-surgery includes radiotherapy and chemotherapy with temozolomide. For DC vaccination as adjuvant therapy, up to now, most of the clinical trial showed no serious vaccine-related adverse events, and it may extend survival [76]. Early insight into tumor lysate-pulsed DC vaccines was provided in a phase III randomized, double-blinded, placebo-controlled clinical trial (*N* = 331) and suggested the addition of the vaccination to standard therapy is feasible and safe [94]. Only 2.1% of patients experienced any grade 3 or 4 adverse events that were at least possibly related to treatment with tumor lysate-pulsed DC vaccines. Autologous DC vaccines benefit patients with malignant glioma but may cause transient and reversible elevation of serum AST/ALT levels [95].

### Peptide modified DC

DC are the most potent professional APCs capable of engulfing foreign antigens from invading pathogens. Through activation of pathogen recognition receptors (PRRs), mature and activated DC via complex downstream signaling pathways (such as cell surface co-stimulatory molecules) assemble the antigen peptide-major histocompatibility complex (MHC) and promote antigen-specific T cell expansion [96]. In early phase I trials, activated DC vaccines showed good safety, but had a weak anti-tumor response, which was not impressive compared with chemotherapeutic regimens. The α-type-1 polarized DC activated by maturation reagents (tumor necrosis factor α, IL 1β, IFN α, IFN γ and polyinosinic acid-polycytidylic acid) and pulsed with cocktail of 5 synthetic peptides (WT-1, HER2, MAGE-A3, and MAGE-A1 or gp100) were well tolerated, except for transient liver dysfunction. Long-term recurrence-free and positive immunological responses were only observed in 1.3% of HLA-A24^+^ glioma patients with stable disease (SD) [97]. The valuable target antigens of immunogenic synthetic peptides that are in existence in tumor lysate still seem to have an advantage to improve the vaccine-induced benefits and relapse-free period and optimal combinations need to be developed.

### Vaccine generated by tumor cells

**Fig. 3:**
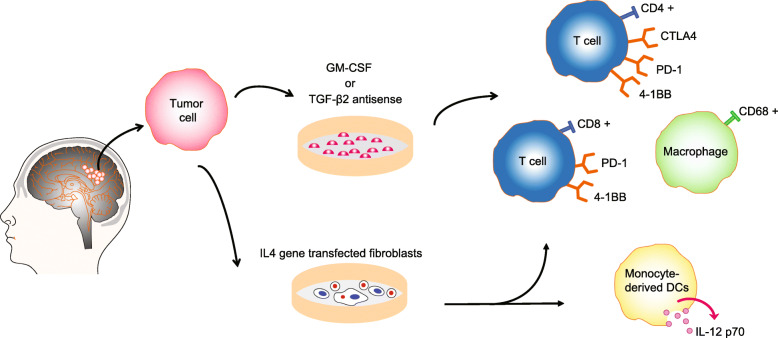
Vaccination using autologous tumor cells in anti-glioma clinical trial Vaccines generated by TAA modified tumor cells with GM-CSF or TGF-β2 induce cellular and humoral anti-tumor immune responses by increasing CD4^+^ T cells with CTLA4, PD-1, 4-1BB, and OX40 expression and CD8^+^ T cells with PD-1 and 4-1BB expression. Using autologous glioma cells and IL-4 gene transfected fibroblasts increases IL-12 p70 level and infiltration of CD4^+^ and CD8^+^ T cells in glioma patients

Vaccination using autologous tumor cells benefit existing humoral and cell-mediated immunity to antigenic epitopes, as well stimulates polyclonal immune attack against multiple, even undetected, TAAs. In separate reports, preclinical evidence shows that tumor cell immunotherapy can enhance anti-glioma immunity and can be effective in intracranial glioma models [98, 99] (Fig. 3). Autologous tumor-derived peptides from GBM can be used to safely immunize patients with recurrent GBM. After vaccination, brain biopsies revealed significant infiltration of focal CD4^+^, CD8^+^, CD56^+^ and IFN γ producing T cells against autologous tumor-derived peptides bound to HSP-96 [100]. Additionally, the NIH Clinical Center provided the promising attempt of administration of tumor lysate vaccine with a potent immune response modifier, such as imiquimod or poly-polyinosinic-polycytidilic acid (ICLC) adjuvant, to stimulate cell-mediated immune responses (Table 2). However, even though the feasibility and safety of vaccination were noted, the relatively weak anti-tumor activity of vaccination with irradiated autologous glioma has been demonstrated in recent clinical trials.

**Table 2:** clinical trial of tumor lysate vaccine in glioma

Malignancy	Phase	Number	Study title	Identifier	Status	Interventions	Observation
Recurrent glioma	I	11	Vaccination with lethally irradiated glioma cells mixed with GM-K562 cells in patients undergoing craniotomy for recurrent tumor	NCT00694330	Completed	Mixed With GM-K562 Cells	Toxicity; PFS; OS
Recurrent HGG	I/II	14	Phase I/II study to test the safety and efficacy of TVI-Brain-1 as a treatment for recurrent grade IV glioma	NCT01081223	Completed	Immune adjuvant plused, activated white blood cells plused	Toxicity; tumor response; PFS; OS; immune responses
Recurrent HGG	II	86	Study to test the safety and efficacy of TVI-Brain-1 as a treatment for recurrent grade IV glioma	NCT01290692	Completed	Immune adjuvant plused, activated white blood cells plused	Toxicity; tumor response; PFS; OS; immune responses
Recurrent grade II gliomas	I	19	Imiquimod and tumor lysate vaccine immunotherapy in adults with high risk or recurrent grade II gliomas	NCT01678352	Completed	Imiquimod adjuvant	T-cell responses; toxicity
DIPG	I	8	Imiquimod/brain tumor initiating cell (BTIC) vaccine in brain stem glioma	NCT01400672	Terminated	Imiquimod adjuvant	Toxicity; tumor response
Newly Diagnosed GBM	I	1	Derivation of tumor specific hybridomas	NCT01702792	Terminated	NA	Toxicity; number of hybridoma clones; production of antibodies
Grade II gliomas	I	30	Neo-adjuvant evaluation of glioma lysate vaccines in WHO grade II glioma	NCT02549833	Recruiting	Poly-ICLC adjuvant	Toxicity; tumor response; PFS; OS; CD4 + and CD8 + T-cell responses; expression of GAAs and APM molecules

*HGG* high-grade glioma; *GBM* glioblastoma; *NA* not applicable; *Poly-ICLC* polyinosinic-polycytidilic acid; *GAA*, lioma-associated antigen; *APM* antigenpresentation machinery. Data collected from clinicaltrials.gov database

### TAA modified tumor vaccine

Following the observance of the effective anti-tumor ability of GM-CSF in vitro and in vivo, the US Food and Drug Administration has approved GM-CSF for use with dose-intensive chemotherapy. A strategy referred to as “GVAX”, composed of GM-CSF-engineered irradiated autologous tumor cells, has demonstrated an immunostimulatory effect related to GM-CSF in extensive preclinical results [101]. Using autologous tumor vaccine plus GM-CSF as an adjuvant, 89% of the vaccinated patients developed an autologous tumor-associated delayed type hypersensitivity (DTH) response; 42% of patients showed radiological evidence of a response, while only 26% patients showed clinical improvement after vaccination [102]. In a phase I study of recurrent malignant glioma (N = 10), irradiated autologous glioma cells were mixed with irradiated K562 cells, which over-expressed GM-CSF, to strengthen its DTH responses and humoral immunity. Vaccination revealed activation of T-lymphocytes, with increasing cytotoxic T-lymphocyte-associated protein 4 (CTLA4), PD-1, 4-1BB, and OX40 expressed CD4^+^ T cells, as well as PD-1 and 4-1BB expressed CD8^+^ T cells. No dose-limiting toxicities were seen when vaccinating patients with subcutaneous and intradermal injections of irradiated autologous glioma cells [103]. Transforming growth factor-beta 2 (TGF-β2) is a secreted protein known as an immunosuppressive molecule, whose dysregulated signaling contributes to the initiation and progression of many cancers, including glioma [104]. Preclinical study demonstrated that inhibition of TGF-β2 expression significantly enhances tumor-cell immunogenicity in eliminating previously implanted tumors [105]. Administration of TGF-β2 modified tumor cells showed recoverable and low-grade treatment-related toxicities and may be safe for glioma patients. In a phase I clinical trial, injection of TGF-β2 antisense-modified autologous tumor vaccine increased survival in some patients and induced generation of cellular and humoral anti-tumor immune responses in stage IV astrocytoma [106].

Vaccination-generated Abs may contribute to the in vivo immunogenicity of selected tumor antigens and may contribute to the therapeutic efficacy of cellular vaccines mainly designed to induce/up-regulate a tumor-specific CTL response. Recently, therapeutic vaccines based on genetically modified allogeneic tumor cells have been evaluated in clinical trial, regarding its advantage of acceptable construction costs, and the host of special antigen presenting. Clinical support for an allogeneic cellular vaccine approach has been validated in melanoma and has shown the extent of immunization against selected tumor antigens [107], but such is not the case in glioma. These findings support the clinical application of autologous tumor vaccines comprised of systemic immunosuppression antigens in glioma. Further randomized trials with allogeneic tumor cell immunotherapy may take a chance to establish efficacy as an alternative approach.

### Fusion of autologous tumor and fibroblast cells

The risk of local recurrence occurs highly at and around the site of injury after surgical removal of the tumor [108]. Previous studies demonstrated fibroblasts may be applicable to cancer-targeting gene therapy for local control of the tumor around the injured tissue [109]. Using autologous glioma cells and IL-4 gene transfected fibroblasts, the feasibility and safety of adjuvant vaccinations were evaluated in patients with newly diagnosed and recurrent GBM, and it demonstrated encouraging immunological and clinical responses without allergic encephalitis [110]. In recurrent GBM, IL-4 dose-dependent infiltration of CD4^+^ and CD8^+^ T cells was observed at local vaccine sites. Vaccinations in HLA-A2^+^ patients demonstrated systemic T cell responses against an HLA-A2-restricted GAA epitope, EphA2883-89. In newly diagnosed GBM, monocyte-derived DC produce high levels of IL-12 p70 followed by two intradermal vaccinations with transfected fibroblasts admixed with DC loaded with autologous tumor lysate. However, detectable IFN γ post-vaccine responses or prolonged progression-free survival was not observed in these participants. To improve the intensity of the vaccine regimen, future studies will need to identify the potential vaccine intensification approach to enhance therapeutic efficacy without detrimental counterbalancing of autoimmunity.

### Autologous natural killer cell therapy

NK cells are highly efficient in the cellular immune response against diseases, including malignancies. The past several years have seen tremendous advances in the selection and expansion of NK cells, and they have been used in clinical trials as adaptive immunotherapy for cancer [111]. Current NK cell-based cancer immunotherapy was designed to improve NK cell paralysis using several approaches, which are more practical for quality control and large-scale production by using stable autologous NK cell lines, for adoptive cellular immunotherapy [112]. In a phase I clinical trial, autologous NK cell therapy was demonstrated to be safe and partially effective in patients with recurrent GBM. The NK cell-rich effecter cells were manufactured from PBMCs through co-culturing with irradiated human feeder cell line (HFWT) and IFN β [113]. To a certain degree, autologous NK cell therapy could not yet exhibit their full cytotoxic capacity in vivo due to MHC class I expression in cancer patients that suppresses autologous NK cells [114]. A previous study demonstrated NK cell cytotoxicity is up-regulated by killer cell immunoglobulin-like receptors (KIRs), which interact with HLA class I ligands [115], while this finding was not tested in phase I clinical trial in human patients with glioma.

## Discussion

Among all the therapies that have demonstrated significant safety and feasibility for gliomas in clinical trials, including radiation, chemotherapy (temozolomide and PCV [procarbazine, lomustine, vincristine]) and targeted therapies (bevacizumab) [116], the impact of cellular vaccine therapies has been most modest in glioma. For unequivocal clinical benefit, improvements should be achieved to maximize the vaccine-induced T cells with optimal amplitude, specificity and effector profile [117]. Combination with immune response modifiers in glioma promises to boost the true power of cellular vaccines and potentially offer long-term protection from tumor recurrence. The cytokine IL-12 is a potent inducer of anti-tumor activity in a variety of preclinical models [118]. By inflammation regulation, IL-12 has been proven to establish a link between innate and adaptive immunity that involves different immune effector cells and cytokines [119]. The inducible expressed IL-12-armored CART cells have broadened the application of CART-based immunotherapy and might provide an alternative therapeutic strategy for cancer treatment [120]. For recurrent glioma, results of a phase I trial have revealed IL-12 increased IFN γ and PD-1 with acceptable tolerability in TILs [121]. Additionally, the U.S. Food and Drug Administration (FDA) has approved synthetic immunomodulatory agents, such as resiquimod and imiquimod, that act as vaccine adjuvants, enhancing cytokine production and skewing immunity towards a Th1/cytotoxic response [122, 123]. Targeting tumor-specific T cells, therapeutic interceptions that inhibit receptors, including PD1 (pembrolizumab, nivolumab), CTLA4 (ipilimumab, tremelimumab) and LAG3 (BMS-986,016), have been approved or are in clinical trials for the treatment of various cancer types [124]. In practice, however, the challenges the BBB poses for glioma therapy and how these immune response modifiers activate the local brain tumor immune system and enhance cytolytic effects should be further discussed in future clinical models.

So far, there is solid evidence that intrinsic factors (sex, age and comorbidities) and vaccine-related factors (adjuvants and vaccination schedule), as well as the immune system (such as innate and adaptive responses), strongly influence vaccine efficacy [125]. Recent progress in the molecular subtypes of histologic-based glioma classification suggests some potential reasons for the indistinctive effort of the cellular vaccine. For example, given the spectrum of aggressive phenotypes, tumor marker-based classification, such as IDH mutation, 1p/19q co-deletion, and TERT promoter mutations, predict favorable prognosis in gliomas [126–128]. In IDH-wild type GBM, a gene-based signature could be a potential prognostic biomarker [129]. Thus, based on molecular markers, educated and advisable vaccine use for a cellular vaccine may be critical for safety and promotable anti-tumor effects in glioma treatment. Typical GBM alterations, such as IDH mutation, NF1 inactivation, and CDK4-MARCH9 locus amplification, characterize tumor-associated immunosuppression [130]. IDH1 mutations caused down-regulation of leukocyte chemotaxis, resulting in reduced immune infiltrates that may contribute, in part, to differences in the aggressiveness of mutant type gliomas [131]. The immune features of B7-H3, an immune checkpoint member found to positively correlate with the grade of malignancy, may become an attractive target for IDH-wild type glioma immunotherapy [132]. These findings encourage researchers to further confirm the tumor response of cellular vaccines based on specific molecular subtypes in the ongoing larger randomized trials, such as IDH1 R132H-DC vaccine (NCT02771301), which may provide the hope to optimize cellular vaccines in gliomas.

To a large extent, cancers are particularly evolutionary events owing to their genetic heterogeneity [133, 134]. An understanding of the heterogeneous characteristics of cancer clones allows us to well address the individual tumor behavior and therapeutic response [135]. Indeed, such a framework could also be applied to explore the patient-specific neoantigens in the course of tumor evolution for successful vaccine immunotherapy. The availability of neoantigen-based personalized vaccines further provides a powerful aid to elucidate inter-individual variability and intra-tumor heterogeneity [136]. In patients with gliomas, intratumour heterogeneity of IDH1 mutations can be considered as a favorable independent prognostic biomarker. Schumacher and colleague demonstrated the promising function of IDH1 mutation–specific vaccination for glioma treatment [137]. In this report, the neopeptides carrying IDH1 R132H p123-142 mutated region were produced to interact with transgenic human MHC-II molecules in glioma mouse model. Peptide vaccination resulted in efficient mutation-specific antitumor immunity in the mouse model with IDH1 R132H-mutated gliomas. The synthetic neopeptides encompassing histone 3 variant H3.3K27M mutation could be recognized by TCR. After then, TCR-transduced T cells specifically lysed the H3.3K27M^+^ glioma cells [138] and prolonged overall survival in patients with diffuse midline gliomas [139]. In addition, Duperret et al. presented a pre-clinical study about utilizing nucleic acid vaccine platform to target tumor multi-neoantigens [140]. These nucleic acid vaccines induced predominantly MHC I-restricted, CD8^+^ T cell responses and subsequently killed tumor cells. Recently, by used personalized neoantigen-targeting vaccines to immunize glioma patients, Keskin et al. found that neoantigen-based vaccines had the potential to evoke the neoantigen-specific CD4^+^ and CD8^+^ T cell responses [141]. Therefore, considering the biological importance of neoantigens in cancer immunotherapies [142], it is reasonable to suppose that the neoantigen-based therapeutic strategies might have a viable future in anti-glioma immune responses.

Up to now, the clinical relevance of immune regulation in glioma research and treatment remains debated. However, emerging studies about the immunological processes participated in glioma tumorigenesis have yielded a basis for clinical translation of glioma-associated vaccination strategies. Even though these immunotherapy strategies have yielded increasingly good outcomes for glioma patients, challenges with vaccine-based immunotherapy still inevitably remain. The first conceptual challenge is the choice of individualized neoantigen to be targeted and the accurate status assessment of such targets [143]. As more and more tumor neoantigens have been identified, the future fields of investigation should focus on the effective neoepitope dosage without tolerization and finding the best neoepitope delivery system [144]. In BALB/c mice, using a potent immunostimulatory adjuvant and delivery system for neoepitope immunization might be of great benefit to induce the CD8 + T cells-mediated tumor rejection response [145]. The immunosuppression and T-cell exhaustion in tumor microenvironment pose additional engineering challenges. Thus, effective treatment of solid tumors with vaccines needs to generate the activated CAR T cells that can function in the immunosuppressive tumor microenvironment [146]. Another apparent limitation is that if used for cancer prevention, vaccines must elicit effective long-term memory in order to avoid causing autoimmunity [147]. In addition, immunotherapy should be carefully considered to be integrated into the existing standard therapies. The immunotherapeutic agents, including vaccine-based therapies, limited its therapeutic potential when used alone [148]. Oppositely, the combination of vaccine therapy with other conventional methods provides obviously synergistic effects on the eradication of cancer cells [149]. Overall, with continued research addressed these limitation, such as clarifying immune-cancer interactions and discovering innovative vaccine targets, we can design novel strategies and technologies to better optimize vaccine-mediated immunity to further improve the outcomes for glioma patients.

## Conclusions

To date, it is well-known that cellular vaccines can be considered as a promising therapeutic strategy for glioma patients. Increasing studies have demonstrated that therapeutic vaccines based on T cells, DC cells, tumor cells and NK cells are feasible and generally well tolerated. More importantly, clarifying the functional roles of cellular vaccines in glioma immunotherapy may bear potential implications for apparent therapeutic advantages and clinical benefits. Even so, in the future, forthcoming investigations are needed to comprehensively explore their unique characteristics involving in anti-glioma immune responses. The in-depth understanding of cellular vaccines will contribute to uncover the detailed mechanisms and biological functions in glioma research and treatment. Furthermore, in view of the non-negligible roles of cellular vaccines, more preclinical and clinical trials can be conducted to focus on their combination with current treatment regimens.

## Data Availability

Not applicable.

## Abbreviations

ADCTA: Autologous dendritic cells/tumor antigen vaccine
CNS: Central nervous system
WHO: World Health Organization
GBM: Glioblastoma
BBB: blood-brain barrier
MGMT: O6-Methylguanine-DNA methyltransferase
Tregs: T regulatory cells
Th1: T helper 1
GAAs: Glioma-associated antigens
TAA: Tumor-associated antigen
CARs: Chimeric antigen receptors
NK: Natural killer
CAR T cell: CAR-engineered T cell
IL13Rα2: Interleukin 13 receptor α2
EGFRvIII: Epidermal growth factor receptor variant III
EphA2: Ephrin type-A receptor 2
HER2: Human epidermal growth factor receptor 2
CTL: Cytotoxic T lymphocytes
PBMCs: Peripheral blood mononuclear cells
CXCL9: C-X-C motif chemokine ligand 9
APCs: Antigen-presenting cells
MFG-E8: Milk fat globule-EGF factor 8
WT1: Wilms tumor protein
gp100: Glycoprotein 100
MAGE: Melanoma-associated antigen
MAGEA3: MAGE family member A3
HLA: Human leukocyte antigen
GM-CSF: Granulocyte-macrophage colony-stimulating factor
KIRs: Killer cell immunoglobulin-like receptors
PD-1: Programmed cell death protein 1
TILs: Tumor-infiltrating lymphocytes
TCR: T cell receptor
HCMV: Human cytomegalovirus
